# From Viral Infection to Autoimmune Reaction: Exploring the Link between Human Herpesvirus 6 and Autoimmune Diseases

**DOI:** 10.3390/microorganisms12020362

**Published:** 2024-02-09

**Authors:** Liba Sokolovska, Maksims Cistjakovs, Asnate Matroze, Modra Murovska, Alina Sultanova

**Affiliations:** 1 Institute of Microbiology and Virology, Riga Stradins University, LV-1067 Riga, Latvia; 2 Faculty of Residency, Riga Stradins University, LV-1007 Riga, Latvia

**Keywords:** HHV-6, human herpesvirus 6, autoimmunity, autoimmune disease, multiple sclerosis, systemic sclerosis, inflammatory bowel disease, autoimmune thyroiditis, chronic fatigue syndrome, DIHS/DRESS

## Abstract

The complexity of autoimmunity initiation has been the subject of many studies. Both genetic and environmental factors are essential in autoimmunity development. Among others, environmental factors include infectious agents. HHV-6 is a ubiquitous human pathogen with a high global prevalence. It has several properties suggestive of its contribution to autoimmunity development. HHV-6 has a broad cell tropism, the ability to establish latency with subsequent reactivation and persistence, and a range of immunomodulation capabilities. Studies have implicated HHV-6 in a plethora of autoimmune diseases—endocrine, neurological, connective tissue, and others—with some studies even proposing possible autoimmunity induction mechanisms. HHV-6 can be frequently found in autoimmunity-affected tissues and lesions; it has been found to infect autoimmune-pathology-relevant cells and influence immune responses and signaling. This review highlights some of the most well-known autoimmune conditions to which HHV-6 has been linked, like multiple sclerosis and autoimmune thyroiditis, and summarizes the data on HHV-6 involvement in autoimmunity development.

## 1. Introduction

Autoimmunity is a highly complex process influenced by the interplay of genetic and environmental factors, so the etiology of many autoimmune diseases remains unclear. Several genetic polymorphisms associated with autoimmunity have been identified, mainly connected with immune responses, e.g., polymorphisms in HLA, cytokine and cytokine receptors, and other genes (TNFAIP3, etc.) [1]. Next to genetic susceptibility, environmental factors are considered equally important [2], and among the most significant ones are infectious agents, especially viruses [3,4].

Human herpesvirus 6 (HHV-6) is among the viruses frequently linked to autoimmune diseases. Since its discovery in 1986 [5], HHV-6 has been divided into two variants. Later, based on distinctive biological, molecular, and epidemiological differences, these variants were designated as two separate species—HHV-6A and HHV-6B [6]. The term HHV-6, used throughout this review and elsewhere, collectively refers to both species. The species seem to have differing associations with autoimmune diseases. However, clear evidence is lacking as many authors fail to differentiate the species, and readily available species-specific serologic tests are absent. The geographical distribution of the two species has been shown to differ as well [6], and seemingly, the disease association of the two species also seems to differ depending on the region, e.g., in the case of autoimmune thyroiditis [7,8].

Several mechanisms of virally induced autoimmunity have been proposed [3,4]. Viral antigen similarity or molecular mimicry can lead to the activation of T and B cells recognizing both self- and non-self-antigens. Researchers have demonstrated molecular mimicry in the case of HHV-6 and multiple sclerosis [9,10]. Another hypothesized mechanism is bystander activation, where an over-active or non-specific antiviral immune response leads to a localized proinflammatory environment, thereby releasing normally sequestered cell antigens. These antigens are subsequently taken up by antigen-presenting cells and can lead to the activation of autoreactive cells. Viral persistence in tissues can elicit a prolonged inflammatory state, with viral antigens constantly stimulating the immune response, leading to a similar condition. Cell antigen release can also occur by lytic infection, which applies to HHV-6 infection [11,12].

HHV-6 possesses several intriguing properties that have led researchers to look into whether this human pathogen could be involved in non-infectious disease development:

- **High prevalence and early primary infection**. The global seroprevalence of HHV-6 ranges up to 95%, although the exact prevalence of each species is unclear [13,14,15]. Primary HHV-6 infection occurs in the first few years of life, with the earliest infection coinciding with the veining of maternal antibody titers [16,17]. It is generally believed that the majority of primary infections are caused by HHV-6B, in many cases associated with the exanthem subitum, an early febrile illness, which in rare cases can lead to encephalitis. HHV-6A is acquired later in life through asymptomatic infection [12].
- **Establishment of latency, with the potential of reactivation and persistent infection**. After the early primary infection, HHV-6, like other herpesviruses, can establish lifelong latency and reactivate. HHV-6 can cause a persistent infection, thus creating a prolonged inflammation due to viral antigens and the immune system’s response to them. Researchers believe HHV-6 mainly establishes latency by integrating into its hosts’ genome [18]. On rare occasions, the integration can be established in gametes, leading to a condition called inherited chromosomally integrated HHV-6 (iciHHV-6) [19]. The exact factors of HHV-6 reactivation are not definitively demonstrated, but reactivation has caused a severe disease burden. Neurological manifestations like encephalitis in immunocompromised individuals who are HIV-positive or have undergone solid organ or hematopoietic stem-cell transplantation have been associated with HHV-6 reactivation [20].
- **Broad cell tropism.** Even though HHV-6 was considered a strictly lymphotropic virus, its cell tropism has turned out to be much broader. Originally the cell entry receptor of both HHV-6 species was thought to be CD46 [21]. CD46 is present in all nucleated cells and is important in complement regulation [22]. Later, it was demonstrated that several strains of HHV-6B did not utilize this receptor and that soluble CD46 could not inhibit the infection. Studies determined that the cell entry receptor primarily used by HHV-B is CD134 [23,24]. Other widely expressed cell surface molecules have also been proposed to be important for HHV-6 entry. These include nectin cell adhesion molecule 2 (CD112) [25] and gp96 (glucose-regulated protein 94) [26]. HHV-6 has been found in cells and tissues with various origins, including immune, endothelial, epithelial, and neuronal, with HHV-6A showing an increased propensity to infect neuronal cells [12,27,28].
- **Immune evasion and modulation mechanisms**. Herpesviruses have long evolved and adapted to ensure a productive infection in their specific host. A large part of their genomes have been devoted to evading and modulating the host immune response. HHV-6 is no exception [29]. The “immunotropic” nature of HHV-6 in itself can be a mechanism of immunomodulation. The infection has been shown to alter immune cell functionality, for example, changing the profile of secreted immune mediators. HHV-6 infection can alter cell surface molecule expression, causing the downregulation of its cell entry receptor CD46, thus dysregulating complement activation, leading to tissue damage. Also, HHV-6 has been shown to increase cytokine levels and create an inflammatory environment [30,31]. Many herpesviruses encode chemokines and chemokine receptors as an immune modulation strategy. HHV-6 encodes a chemokine (U83) and two chemokine receptors (U12 and U51) [32]. Both of the chemokine receptors are quite poorly studied. However, studies have shown they bind a variety of human chemokines CCL2, 3, 4, 6, and others. In the case of the chemokine RANTES (CCL5), U51 has been shown to downregulate its expression and secretion [32,33]. A role in viral replication and possibly cell-to-cell transmission has also been demonstrated for U51 [34]. Additionally, U12 and U51 are putative G-protein couple-receptor (GPCR) homologs. They possess similarities with human CCRs not only in functionality, but on a molecular level as well. The ability to spread cell-to-cell is an immune evasion strategy since this mode of transmission could allow the virus to be practically invisible to the immune system and persist in solid tissues [35].

**Figure 1:**
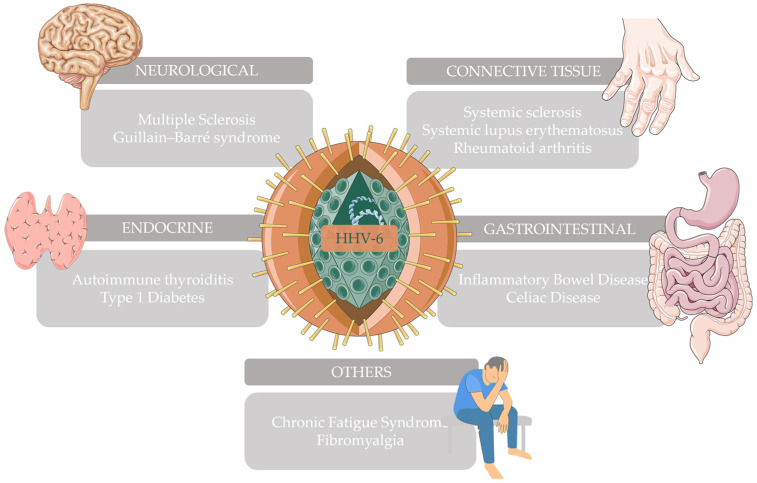
Autoimmune diseases that have been associated with HHV-6 and have been further explored in this narrative review. This figure was partly generated using Servier Medical Art (https://smart.servier.com/ accessed on 28 December 2023) and Vecteezy (https://www.vecteezy.com/ accessed on 28 December 2023).

This narrative review explores how HHV-6, possessing all the properties described above, has been associated with various well-known neurological, connective tissue, gastrointestinal, endocrine, and other autoimmune diseases (Figure 1).

## 2. Autoimmune Neurological Diseases

Autoimmune disorders of the nervous system may affect any part of the nervous system, both the central and peripheral nervous systems. These debilitating disorders lead to neurodegeneration and motor as well as sensory loss. Since HHV-6 has been known to infect cells of a neuronal origin [27,28], the association between autoimmune neurological diseases and HHV-6 has been extensively studied.

**Multiple sclerosis** is the most common immune-mediated inflammatory demyelinating disease of the central nervous system. Viral involvement in multiple sclerosis (MS) has long been suspected, and studies have explored the connection with the Epstein–Barr virus (EBV), endogenous retroviruses, and others [36,37]. Since the mid-1990s, studies have accumulated evidence suggesting the association of HHV-6 with MS. HHV-6’s “footprints”—DNA, RNA, and proteins—have frequently been found in MS patient samples. A recent comprehensive review has summarized studies documenting the association between HHV-6 and brain diseases (MS being among them) and potential mechanisms of involvement [38].

Several studies have demonstrated that HHV-6 nucleic acids and proteins can be detected in higher abundance in MS plaques, especially in oligodendrocytes and microglia, compared to other parts of the brains of MS patients, brains of patients with other neurological conditions, or brains of individuals who died of unrelated causes [39,40,41,42,43,44,45,46]. While a few studies fail to demonstrate this [47,48], the vast majority confirm the association of HHV-6 nucleic acids and antigens with MS plaques. Additionally, several studies have found markers of HHV-6 more frequently in the cerebrospinal fluid, plasma, and peripheral blood mononuclear cells (PBMCs) of MS patients [49,50,51,52,53,54]. Recently, a study by Domínguez-Mozo et al. found HHV-6 miRNAs in MS patient serum and CSF samples, which could aid the maintenance of HHV-6 latency [55]. The amount of HHV-6 nucleic acids and active infection markers in diseased tissue or blood have been shown to correlate with the exacerbation of the disease and relapsing episodes [56,57,58,59].

Differences in the immune response against HHV-6 have also been observed in MS patients. Higher levels of HHV-6-specific IgM antibodies have been demonstrated, while the levels of IgG antibodies were not always found to be higher compared to healthy individuals [54,60,61,62,63]. A study following MS patients treated with disease-modifying therapy found that patients with a decrease in anti-HHV-6 IgG antibodies were more likely to be free of relapsing episodes and disease progression when compared to patients whose titers had increased [64]. Additionally, a large study found that antibodies against HHV-6A (and not HHV-6B) early protein (IE1) were positively associated with MS risk [65]. A proinflammatory environment with elevated levels of TNF-α, IFN-γ, IL-1β, IL-6, IL-12, and CCL-5 has also been demonstrated in MS patients seropositive for HHV-6 [59,66].

Since other viruses have also been implicated in MS, studies have investigated neurotropic viruses’ interplay and suggested that HHV-6 could transactivate EBV [67,68,69]. The molecular mimicry phenomenon has also been demonstrated in the context of MS. HHV-6 membrane protein U24 shares identical amino acid sequences with myelin basic protein, and studies have shown that MS patients have a higher frequency of circulating cross-reactive T cells in comparison to healthy controls [9,10]. Further studies of HHV-6A U24 showed that it could be implicated in demyelination by binding the brain enzyme Nedd4 [70].

To summarize, HHV-6 (most likely HHV-6A) could be involved in MS pathology through various pathways. These include neuroinflammation (either by itself or by transactivating EBV or other neurotropic viruses), demyelination (through molecular mimicry and other characteristics of U24), and impaired remyelination (by direct oligodendrocyte infection) (Table 1).

**Guillain–Barré syndrome (GBS)** is a rapid-onset muscle weakness caused by the immune system damaging the peripheral nervous system. Infectious agents have been suggested to be relevant triggers, and approximately 75% of patients show signs of infections shortly preceding diagnosis of GBS [71]. However, the evidence of this infectious agent being HHV-6 is sparse and inconclusive, and most studies have analyzed a fairly small number of samples. The most recent study analyzed serum and cerebrospinal fluid samples from 14 GBS patients and found only one positive serum sample. The positive patient was in relapse at the point of sampling [72]. Detection of HHV-6 in cerebrospinal fluid could indicate that the virus is actively replicating within the CNS, although exceptions exist. In people with iciHHV-6, HHV-6 presence in CSF does not always indicate neurologic disease [73]. One study demonstrated that 1/3 of GBS patients’ cerebrospinal fluid samples harbored HHV-6 [74]. Another showed the presence of HHV-6A in 4/14 patient samples, coincidental with clinical signs [75]. Detecting active HHV-6 infection in the CNS during the clinical course of GBS might be linked with GBS development. This could be true not only for infections that precede GBS, but also for infections that happen at the same time as the disease. Another potential HHV-6 association with GBS was illustrated by a case report where an infant developed GBS 20 days after exanthem subitum, which was serologically confirmed to be associated with an HHV-6 infection [76]. Also, case reports have documented the occurrence of GBS following HHV-6 reactivation in hematopoietic stem cell transplantation [77,78]. At the moment the evidence for HHV-6 and GBS is insufficient to draw conclusions, but more research is needed to elucidate the association and possible mechanisms.

## 3. Autoimmune Connective Tissue Diseases

Autoimmune connective tissue diseases include such conditions as systemic sclerosis or scleroderma (SSc), systemic lupus erythematosus (SLE), and rheumatoid arthritis (RA). They cause chronic inflammation that affects the connective tissues, skin, joints, and many other organs and systems. The etiology of these diseases remains unclear, but research findings suggest several viral infections may be involved. Reactivation of HHV-6 has been detected in several of the aforementioned conditions, and steroid treatment, which is often used to treat these patients, has been associated with HHV-6 reactivation [79,80]. Research has suggested that HHV-6 may predispose patients to the development of autoimmune connective tissue diseases or, conversely, that these disorders may predispose patients to HHV-6 reactivation [81,82].

**Systemic sclerosis** is a chronic multisystem disease characterized by widespread vascular dysfunction and progressive skin and internal organs fibrosis, causing ischemic and fibrotic tissue damage, cutaneous sclerosis, digital ulcers, and pulmonary, cardiac, gastrointestinal, and/or renal involvement. Numerous infectious agents have been suggested as possible causative factors of SSc. Among viruses, those that persist and may reactivate, such as human cytomegalovirus, HHV-6, and parvovirus B19, could be considered the main candidates.

A recently published review on the role of environmental factors in the etiopathogenesis of SSc has summarized the possible role of HHV-6 in SSc [83]. First, active HHV-6 infection (presence of viral DNA in the sera) has been detected in SSc patients, and HHV-6 in the serum was more frequently found in patients with active disease [81,82]. HHV-6 has been shown to infect endothelial cells and cause them to lose their angiogenic abilities [84]. It also alters the expression of HLA-G, which mediates angiogenesis inhibition, and has been shown to be increased in SSc patients [85]. Also, KIR2DL2, an NK cell inhibiting molecule recognized as a factor of impaired anti-herpesvirus immune response, has been associated with a higher risk of developing SSc. Significantly higher prevalence and HHV-6 load were found in SSc patients’ PBMCs, and all analyzed skin biopsy samples contained HHV-6A. Additionally, impaired NK cell activity was found in the presence of higher HHV-6 loads [86].

In vitro studies have revealed that HHV-6 (mainly HHV-6A) can induce the expression of pro-fibrotic factors in endothelial cells. Also, HHV-6A can modulate the expression of pro-fibrotic and pro-apoptotic factors and fibrosis-associated miRNA in fibroblasts [87,88]. Altogether, the accumulated research suggests that HHV-6, particularly HHV-6A, may play a role in SSc onset or progression.

**Systemic lupus erythematosus** is a chronic autoimmune disease of unknown etiology that can affect practically any organ. The production of several antinuclear antibodies is a staple of this disease. Patients with SLE can present with a wide range of symptoms, from mild joint and skin involvement to life-threatening renal or central nervous system involvement [89]. Infections have been suggested as causal factors for SLE, but results are still inconclusive. For example, EBV seropositivity is higher in adults and children with SLE than in age-matched controls, but no conclusive data have established that EBV infection influences the future risk of SLE [90]. Similarly, inconclusive results are gathered for HHV-6. HHV-6 DNA has been found in SLE patient samples, and viremia has been found more frequently in patients with active disease [81]. Some have detected other herpesviruses, not HHV-6 [91,92,93]. Reactivated HHV-6 infections have been found to be frequent in SLE [94]. Regarding HHV-6-specific antibody responses, studies report no differences between SLE patients and healthy controls [95]. The available data now strongly support the involvement of EBV in SLE pathogenesis. However, more research is necessary since markers of HHV-6 infection can be found in samples from SLE patients.

**Rheumatoid arthritis** is a chronic inflammatory disease mainly affecting joints but which can also involve other organs. It typically leads to deformity by destroying joints through the erosion of cartilage and bone. For a long time, viral infections have been suspected to have an important role in the etiology and pathogenesis of RA, either through direct joint tropism that causes tissue damage or their ability to activate immune responses directed at joint tissues [96]. Few studies have investigated HHV-6 in RA patients. A’lvarez-Lafuente et al. showed a significant difference in the prevalence of HHV-6 DNA and viral load in RA patients’ serum samples compared to controls, with viral presence in cell-free serum indicating active infection. The same study found HHV-6 DNA in RA patient PBMCs, but without significant differences compared to healthy controls [97]. Another study showed similar findings. A significantly higher presence of HHV-6 DNA was found in the serum of patients with autoimmune connective tissue disease, including RA. However, no significant difference in HHV-6 viral load was observed between sera of autoimmune connective tissue disease patients and controls [82]. Another study demonstrated that HHV-6 DNA could be detected in synovial tissues and fluid, and antigens could be visualized in synovial tissues of RA patients by immunohistochemistry, albeit the tissues analyzed were from a small number of RA patients. The same study demonstrated some connection between HHV-6 infection and RA disease activity [98]. Conversely, another study analyzing RA patients’ synovial fluid could not detect HHV-6 DNA [99]. The main problem in HHV-6 and RA studies is the lack of research investigating joint tissue samples; therefore, it is difficult to speculate on the possible direct involvement of this herpesvirus in RA development.

## 4. Autoimmune Gastrointestinal Diseases

Autoimmune gastrointestinal diseases are diverse, often debilitating conditions characterized by autoimmunity and immune-mediated injury [100]. They include such conditions as celiac disease (CD) and inflammatory bowel disease (IBD), which encompasses Crohn’s disease and ulcerative colitis. These conditions are thought to be on the rise since many developing countries are becoming more westernized. The global prevalence of these conditions is estimated to surpass 0.3 per 100,000 individuals [101].

As with other autoimmune conditions, infections are listed among potential environmental factors in their pathogenesis [102,103]. Most often, the listed pathogens include viruses such as rotavirus, which are strictly thought of as gastrointestinal pathogens. However, HHV-6 may also be considered. Even though HHV-6 is mainly associated with skin and neurological manifestations, gastrointestinal symptoms following primary infection, later exposure, or reactivation have been described [16,104]. Moreover, HHV-6 has been detected in gastrointestinal tissues, such as the small and large intestine and stomach [27].

**Inflammatory bowel disease** is a chronic immune-mediated inflammatory disease characterized by an inflammation of the gastrointestinal tract. It encompasses both Crohn’s disease and ulcerative colitis. Several infectious agents (both bacterial and viral) have been investigated as potential contributors to disease development or exacerbation. A recent systematic review summarized the pathogens linked with IBD; many herpesviruses like EBV, CMV, and HHV-6 were among them [105]. Sipponen et al. investigated ileocolonic biopsies from both Crohn’s disease and ulcerative colitis patients for HHV-6. The study found that nearly half of the IBD patient biopsies harbored HHV-6B antigens. The non-IBD control biopsies harbored HHV-6B at a similar frequency, but the viral antigen expression intensity was lower [106]. Shimada et al. reported similar gastrointestinal sample HHV-6 positivity [107], yet others found HHV-6 in less than 10% of IBD patient colonic samples [108,109]. Sipponen et al. also reported that HHV-6B antigen expression intensity significantly correlated with endoscopic disease severity and histologic intensity in several sites. Interestingly, it was shown that coexpression of HHV-6B and CMV antigens was associated with endoscopic disease severity [106]. Several studies have investigated the significance of combined herpesvirus infection and found that it increases the risk of colectomy in IBD patients [108].

**Celiac disease** is characterized by sensitivity towards gluten and subsequent immunological intestinal injury. Infections are hypothesized to be connected with CD development via the enhancement of intestinal permeability, leading to increased gluten passage across the mucosa or via molecular mimicry with gluten [102]. Frequent rotavirus infections have been shown to increase the risk of CD development, and the introduction of rotavirus vaccination has shown a protective effect [110]. When it comes to HHV-6, only one study has shed light on the association between HHV-6 and CD. The study analyzed 40 CD patients’ intestinal biopsies and found HHV-6B in 63% of the biopsies, but a similar detection rate was observed in non-CD individuals. Interestingly, HHV-6A was detected in neither of the groups’ biopsies [111]. While it seems that HHV-6 is not directly involved in acute CD, its potential role in disease onset remains unknown. Although direct evidence linking HHV-6 and CD has not been found, studies exploring the increase in the risk of CE development following HHV-6 primary infection, especially in those children experiencing gastrointestinal symptoms, have not been conducted. Such studies could help elucidate whether HHV-6 is linked with the onset of CD.

## 5. Autoimmune Endocrine Diseases

The human endocrine system has been plagued by various autoimmune disorders, varying in severity, morbidity, and treatability. Patients suffering from these disorders are destined for lifelong hormone replacement therapy. The link between HHV-6 and two of the most common autoimmune endocrine disorders—type 1 diabetes mellitus and autoimmune thyroid diseases (AITD)—will be further described.

**Autoimmune thyroid diseases** are the most prevalent autoimmune disorders affecting 0.2 to 1.3% of the general population of the iodine-sufficient parts of the world, and are the most frequent conditions affecting the thyroid. Nevertheless, the autoimmunity-triggering mechanisms remain unclear [112,113,114]. The AITD spectrum includes two main presentations of the disease—Graves’ disease and Hashimoto thyroiditis—although strict lines between the two cannot be drawn as patients have been described to progress from one to the other [115]. Several viruses have been implicated in AITD development, including HHV-6 [116,117]. While some studies have shown inconclusive or no evidence for HHV-6 association with AITD [118,119,120], later research encompassing more comprehensive investigations seems to favor this connection and provide substantial results to support this.

A comprehensive study from 2012 provided strong virologic and immunologic evidence linking HHV-6A and autoimmune thyroiditis development. This study demonstrated a higher HHV-6 DNA prevalence in thyroid tissues from Hashimoto thyroiditis (HT) patients compared to controls (82% vs. 10%). Diseased thyroid samples were also shown to harbor higher viral loads and active infection markers. The few HHV-6 positive control group samples harbored latent infection. Since lymphocytic infiltration is a hallmark of autoimmune thyroiditis and HHV-6 has a propensity to infect lymphocytes, this study also analyzed which cells harbored HHV-6. The analysis revealed that HHV-6 was mainly detected in the epithelial cell fractions (the thyrocytes), not the infiltrating lymphocytes, as illustrated by higher viral loads and active infection. The tropism of HHV-6 for thyroid cells was further verified by in vitro thyrocyte culture experiments. The experiments demonstrated that thyrocytes are indeed permissive to HHV-6 infection. It was also demonstrated that HHV-6 induces the expression of HLA-II molecules in thyrocytes, thus enhancing their antigen-presenting capabilities. Additionally, immunological studies revealed that HHV-6 infected thyrocytes became more susceptible to NK-mediated killing, that NK cells derived from HT patients are more aggressive towards HHV-6 infected thyrocytes, and that these patients have an increased number of virus-specific T cells [7]. More recently, the same group further explored the connection between HHV-6A and autoimmune thyroiditis by analyzing miRNAs. The study found that in vitro HHV-6A infection of human thyrocytes and T cells induces the modulation of miRNAs associated with AITD development. Notably, the modulation pattern coincided with the one observed in vivo in AITD patients [121]. A study from Iran also demonstrated the association of HHV-6A and AITD by increased viral detection in samples from HT patients, although only serum samples were used [122].

Our work has also demonstrated similar results. Over 90% of AITD thyroid glands contained HHV-6 DNA, more frequently displaying the presence of active infection markers and viral antigens, as illustrated by immunohistochemistry and immunofluorescent microscopy [8]. We have also demonstrated that AITD patients have lower levels of the aforementioned chemokine RANTES. This is especially true in patients with active infection markers (among them the virus-encoded chemokine receptors U12 and U51), pointing to the possible role of viral chemokine receptors in the autoimmunity process [123]. An immunologic study exploring the HHV-6 encoded chemokine receptors found that AITD patients harbor antibodies against U12 and U51-derived peptides, highlighting that immune response against these proteins could play a role in disease development or exacerbation [124]. Interestingly, we have only been able to detect HHV-6B in our AITD patient thyroid samples. This fact was confirmed by E. Caselli and group, the authors of the 2012 study [7,8]. The differing species associations further illustrate the complexity of HHV-6 and the need for further research to elucidate how HHV-6 influences autoimmunity development and whether it differs among the species.

Overall, it seems that both HHV-6 species could contribute to AITD development or exacerbation in various ways. Either through direct thyrocyte infection that causes significant changes in the cells, through immunological modulation (HLA-II expression, increased NK-mediated killing, chemokine level changes), or through direct immune responses against HHV-6 (HHV-6 specific T cells, HHV-6 protein-specific antibodies) (Table 2).

Recently, several studies have been published exploring the association between HHV-6 and **type 1 diabetes (T1D)**. T1D is a well-known autoimmune condition characterized by the immune-mediated destruction of insulin-producing beta cells. Much like any other autoimmune disease, several infectious agents have been implicated, especially enteroviruses. A study from 2017 found HHV-6B in pancreatic tissues of both diabetic and nondiabetic patients. These researchers demonstrated the presence of latent HHV-6B, with higher viral loads in the pathology-associated islets compared to the surrounding exocrine tissue. Although the study analyzed a relatively small number of samples and found HHV-6 in both diabetic and nondiabetic samples, it hypothesizes that a short episode of HHV-6 reactivation in the islets could trigger an immune response that could aid in the destruction of the cells [125]. A recent 2020 study, built on the results previously reported, demonstrated a higher expression of HHV-6 glycoprotein gB in the islets of T1D patients and the presence of active infection. However, a correlation between HHV-6 protein expression and other T1D markers (CD8 T cell infiltration and MHC I expression) was not found. Also, the study did not differentiate the detected HHV-6 species. Researchers agree that further studies are necessary and doubt that HHV-6 is the primary cause of T1D, but believe that the role of HHV-6 in T1D may be indirect by enhancing the autoimmune process already taking place [126].

## 6. Other Autoimmune Diseases

HHV-6 has also been linked with several conditions whose etiology is thought to involve autoimmune processes, such as Myalgic Encephalomyelitis/Chronic Fatigue Syndrome (ME/CFS) and fibromyalgia (FM).

**Myalgic Encephalomyelitis/Chronic Fatigue Syndrome** is a debilitating disease characterized by persistent fatigue, post-exertional malaise, cognitive impairment, and chronic pain, among other symptoms. The presence of autoantibodies and other markers in patients with ME/CFS has led researchers to believe that the disease could be autoimmune in nature [127]. Various pathogens have been investigated, but persistent viral infections have garnered the most attention from researchers [128]. A link between HHV-6 and ME/CFS was suggested as early as 1988 [129]. While there are studies that question the link between HHV-6 and ME/CFS [130,131,132], many more give strong evidence of the association. Several studies have demonstrated that anti-herpesvirus drugs benefit patients with ME/CFS. Treatment with valganciclovir improved patient mental and cognitive functioning [133,134].

A study from 2012 found that active HHV-6 infection and coinfection with other herpesviruses were more common in the 108 ME/CFS patients investigated. Chapenko et al. also demonstrated that active infection correlated with the occurrence of the clinical symptoms of ME/CFS [135]. In a recent study, Gravelsina et al. provided supportive evidence for the potential association of HHV-6 and ME/CFS and the disease’s autoimmune nature. The study investigated 134 ME/CFS patient serum samples and demonstrated that a higher HHV-6 load was associated with more severe disease and higher HHV-6 loads correlated with higher muscarinic acetylcholine receptor autoantibody levels [136]. A seminal study by Kasimir et al. addressed a crucial shortcoming regarding research into HHV-6’s association with various diseases. While commonly tested biological fluids like blood are less invasive for the patient and more comfortable for the researchers, they can often lack the signs of viral activity, especially since HHV-6’s link with autoimmune conditions is thought to be associated with tissue-restricted viral reactivation. The study investigated postmortem brain biopsies of ME/CFS patients and controls. It demonstrated abundant HHV-6 miRNA expression (indicating active infection) exclusively in ME/CFS patients’ neuronal tissues. Additionally, markers of active HHV-6 infection were found in regions of the brain linked with several of the symptoms of ME/CFS [137].

An interesting link between ME/CFS and herpesviruses could be deoxyuridine triphosphate nucleotidohydrolase (dUTPase) proteins. dUTPases are encoded by humans and several herpesviruses (e.g., HHV-6 and EBV). Herpesvirus dUTPases have immunomodulatory properties and interact with toll-like receptors; thus, researchers believe these proteins could be important in herpesvirus-associated disease pathophysiology [138]. Studies have demonstrated that ME/CFS patients harbor antibodies against herpesvirus dUTPases, sometimes accompanied by human dUTPase antibodies [139]. Further studies by the same group demonstrated elevated serum levels of activin A and IL-21, which correlated with herpesvirus dUTPase antibodies. These signaling molecules affect T follicular helper cell differentiation; thus, the researchers hypothesize that these herpesvirus proteins could lead to abnormal germinal center and extrafollicular antibody responses, potentially leading to autoantibody secretion [140].

Mitochondrial dysfunction is thought to be an essential part of the development and progression of ME/CFS [141]. A study exploring the effects of HHV-6A reactivation on mitochondria demonstrated that HHV-6A induced mitochondrial fragmentation and the inhibition of several proteins important in amino and fatty acid oxidation and glucose metabolism in vitro. Furthermore, the same study demonstrated similar mitochondrial changes in vitro when ME/CFS patient sera were adoptively transferred [142].

In summary, HHV-6 seems like a likely environmental factor in ME/CFS since both immune responses against HHV-6 and markers of infection have been found in patients. Additionally, studies have proposed potential mechanisms for how HHV-6 could be involved in the development of ME/CFS—through direct infection of the brain, mitochondrial fragmentation, and others (Table 3).

**Fibromyalgia**, much like ME/CFS, remains an etiologically mysterious condition. FM is characterized by chronic, widespread pain, but patients may also experience fatigue, sleep disturbances, and cognitive impairment. While the autoimmune nature of this disease is under heated debate, such findings as GPCR autoantibodies and small fiber neuropathy seem to support it [143]. FM can occur together with other chronic pain conditions or could be triggered by environmental stressors. As in ME/CFS, infections are considered as potential triggers. To date, only one study has explored the association of HHV-6 with FM. The 2019 study analyzed whole blood and plasma samples from 43 FM patients for the presence of HHV-6, collected information on symptoms, and performed sensory testing to assess nerve fiber damage. FM patients were shown to harbor HHV-6 DNA in both whole blood and plasma more frequently, with the latter indicating an active infection. Additionally, HHV-6 infection correlated with nerve fiber damage [144]. Although the evidence is lacking now, the research interest in fibromyalgia is on the rise both in the field of pathogenetic mechanisms and better diagnostics, with much of this new attention deriving from the observations of FM following COVID-19 and similarities between FM and post-COVID syndrome [145].

## 7. DIHS/DRESS and Autoimmune Diseases

Another intriguing link between HHV-6 and autoimmune diseases is **Drug-induced hypersensitivity syndrome (DiHS)/drug reaction with eosinophilia and systemic symptoms (DRESS)**. This condition is characterized by severe multiorgan hypersensitivity reactions caused mainly by a limited number of eliciting drugs in patients with a genetic predisposition. Symptoms of this condition can include fever, widespread rash, facial edema, organ involvement (most commonly the liver), and hematological abnormalities, including eosinophilia and atypical lymphocytosis. Certain types of drugs have been repeatedly documented as culprits in DiHS/DRESS-anti-convulsants, anti-microbial agents, antiviral agents, antipyretic agents, and others [146,147].

A hallmark of this condition is herpesvirus reactivation, especially HHV-6. A recent review revealed that among the analyzed articles, HHV-6 reactivation was significantly more common than other herpesviruses and affected 63% of DRESS patients [148]. The reactivation of HHV-6 has been strongly associated with DiHS/DRESS severity and relapsing episodes following the initial acute phase, even after the interruption of the culprit drug [148,149].

The onset of new autoimmune diseases has been reported among the sequelae of DIHS/DRESS, such as autoimmune thyroiditis, systemic lupus erythematosus, autoimmune hemolytic anemia, reactive arthritis, and others [146,147]. A study analyzing 52 DRESS patients revealed that autoimmune sequelae were associated with higher rates of HHV-6 reactivation [150]. The link between DIHS/DRESS, HHV-6, and autoimmunity seems especially strong in the case of thyroiditis. Several cases of thyroiditis development have been documented following DiHS/DRESS and HHV-6 reactivation [151,152,153,154]. The exact mechanism of how HHV-6 reactivation in DIHS/DRESS leads to downstream autoimmune reactions is highly complex and remains unclear. However, it is thought to be linked with HHV-6 mediated depletion of T regulatory cells, leading to immune dysfunction and the heightened immune response towards HHV-6 infected cells, leading to tissue damage [148].

## 8. Conclusions

The emerging interest in the role of environmental factors in autoimmune disease development has identified both newly discovered and long-known pathogens as potential triggers or exacerbators of autoimmunity [3,155]. Viruses with certain properties like broad cell tropism, establishment of latency, and persistence have been especially focused on. HHV-6 qualifies as a candidate as it possesses all these characteristics, like a broad spectrum of immune evasion and immunomodulation tactics. Most hypothesized mechanisms of viral involvement in autoimmunity, like molecular mimicry and viral persistence, have been demonstrated for HHV-6.

However, more research is necessary, especially in autoimmunity-affected tissues, as tissue-specific reactivation without active HHV-6 infection in blood has been documented in autoimmune and other conditions [8,86,123,156,157]. More research is also necessary to elucidate the roles of specific HHV-6 proteins, which are still poorly understood. These proteins could potentially affect the immune processes in the host, for example, the virus-encoded chemokine receptors, which are GPCRs. Recently, GPCR autoantibodies have been shown to be pathologically significant [158]. Additionally, much remains unclear about HHV-6 species-specific autoimmunity triggering mechanisms. Does each species exhibit its own unique mechanisms? Or does detecting a specific HHV-6 species in autoimmune patient samples rely only on the virus’s geographic distribution?

Much remains to be perfected when it comes to the research methodology. Not all studies indicate the species of HHV-6 being investigated—if they did, this may help answer the question posed in the previous paragraph. It is also worth mentioning that a limitation to many of the studies discussed in this review could be that the widely used detection of viral transcripts may not directly mean protein expression. More studies on viral proteomics are necessary, as proteins are both immunomodulators and possible antigens. Also, discussing or determining iciHHV-6 in a particular study would greatly aid the legitimacy and clinical significance of the obtained results.

Some of the contradictory or inconclusive studies mentioned in this review could indicate that HHV-6 might play a part role and be merely a puzzle piece in the big mosaic of autoimmunity. Therefore, it is important to study all the possible HHV-6 mechanisms by which the virus could be implicated in autoimmunity development and the interplay between HHV-6 and other environmental and/or genetic factors.

The post-COVID-19 era might bring about a renewed interest in research targeting herpesviruses and HHV-6 in particular, as studies have reported herpesvirus reactivation in COVID-19 patients [159]. Additionally, early in the pandemic, researchers reported the presence of well-known autoantibodies and the incidence of autoimmune diseases following COVID-19 [155]. Could herpesviruses be linked with COVID-19 and autoimmunity onset thereafter? Many more studies are necessary to answer this question, but the similarity of long-COVID with ME/CFS and the presence of herpesvirus reactivation in long-COVID patients may be a start to answer this question and sway the answer to support herpesvirus involvement [160].

For a long time, HHV-6 has been thought of as mainly a benign bystander, causing illness in the young or the immune suppressed. However, the years of research and the accumulated links with a plethora of autoimmune diseases should change how this virus is perceived. HHV-6 should be viewed not only as a bystander but as a clinically relevant pathogen capable of immune dysregulation leading to disease.

Overall HHV-6 possesses characteristics of a virus of autoimmunity. Studies have demonstrated the relevance of HHV-6 in the context of autoimmunity. It has been detected in autoimmunity-affected tissues and has been shown to infect autoimmunity-relevant cells, changing their functionality or lysing the cells. It has also been shown to promote an inflammatory state and alter immune responses. Although much has been studied, more research is necessary to undoubtedly consolidate HHV-6 as a virus of autoimmunity.

## Figures and Tables

**Table 1:** Main findings and potential mechanisms linking HHV-6 with MS. CSF—cerebrospinal fluid; PBMC—peripheral blood mononuclear cells; IE1—immediate early protein 1; MBP—myelin basic protein. The table was partly generated using Servier Medical Art (https://smart.servier.com/ accessed on 28 December 2023) and Vecteezy (https://www.vecteezy.com/ accessed on 28 December 2023).

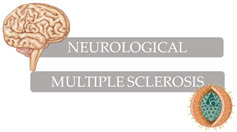	↑ HHV-6 infection markers in plaques [39,40,41,42,43,44,45,46]
	↑ HHV-6 infection markers in CSF, sera, PBMC [49,50,51,52,53,54,55]
	↑ HHV-6 load → Exacerbation, Relapse [56,57,58,59]
	↑ HHV-6 IgM, IgG [54,60,61,62,63]
	↓ HHV-6 IgG → ↓ Relapse episodes [64]
	↑ HHV-6A IE1 IgG → ↑ MS risk [65]
	↑ TNF-α, IFN-γ, IL-1β, IL-6, IL-12, CCL-5 in HHV-6 Ig (+) [59,66]
	Molecular mimicry HHV-6 U24 and MBP [9,10]
	↑ Cross-reactive U24/MBP T cells and antibodies [9,10]
	U24 and Nedd4 → Demyelination [70]
	HHV-6 transactivates EBV [67,68,69]

**Table 2:** Main findings and potential mechanisms linking HHV-6 with AITD. vGPCR—viral G-protein coupled-receptor. The table was partly generated using Servier Medical Art (https://smart.servier.com/ accessed on 28 December 2023) and Vecteezy (https://www.vecteezy.com/ accessed on 28 December 2023).

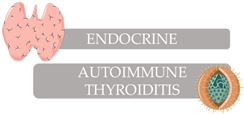	↑ HHV-6A and B infection markers in thyroid, sera [7,8,122,123]
	↑ HHV-6A and B active infection in thyroid [7,8,123]
	Thyrocytes permissive to HHV-6 [7,121]
	↑ HHV-6 markers in thyrocytes [7]
	HHV-6 in thyrocytes → ↑ HLA-II expression [7]
	HHV-6 in thyrocytes → ↑ NK-mediated killing [7]
	↑ HHV-6 specific T cells [7]
	HHV-6 in thyrocytes → ↑ AITD miRNAs [121]
	↓ RANTES (CCL-5) and HHV-6 vGPCRs [123]

**Table 3:** Main findings and potential mechanisms linking HHV-6 with ME/CFS. PBMC—peripheral blood mononuclear cells; anti-M4—muscarinic acetylcholine receptors (M4 AChR); dUTP—deoxyuridine triphosphate nucleotidohydrolase; AA—amino acid; FA—fatty acid. The table was partly generated using Servier Medical Art (https://smart.servier.com/ accessed on 28 December 2023) and Vecteezy (https://www.vecteezy.com/ accessed on 28 December 2023).

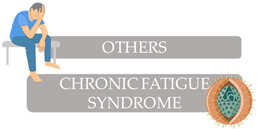	↑ HHV-6 infection markers in sera, PBMCs [128,135,136]
	HHV-6 active infection and Clinical symptoms [135]
	↑ HHV-6 load in sera → ↑ Disease severity [136]
	↑ HHV-6 load in sera → ↑ anti-M4 Abs [136]
	↑ HHV-6 active infection in brain [137]
	↑ HHV dUTPase antibodies [139]
	HHV dUTPase antibodies → ↑ activin A, IL-21 [140]
	HHV-6A reactivation → Mitochondrial fragmentation [141]
	HHV-6A reactivation → ↓ AA, FA, glucose metabolism [142]
